# Disentangling risk of progression and clinical trajectories in preclinical AD: views from the International Working Group

**DOI:** 10.1002/alz70856_098966

**Published:** 2025-12-24

**Authors:** Nicolas Villain

**Affiliations:** ^1^ Assistance Publique Hopitaux de Paris (APHP), Paris, France

## Abstract

**Background:**

The International Working Group (IWG) framework distinguishes two nosological categories within the cognitively unimpaired biomarker‐positive population: “presymptomatic Alzheimer's disease (AD)” (high risk of progression) and “asymptomatic at risk” (low risk)^1^, unlike the Alzheimer's Association (AA) criteria, which classify both under “preclinical AD.”^2^ This stratification underscores the importance of clinical risk, as opposed to the pathophysiological continuum, for research on biomarkers and therapies.

**Method:**

This review synthesizes findings from the literature, highlighting the IWG and AA frameworks concepts and emphasizing the IWG framework significance on 1) clinical and biomarker trajectories and 2) tailored pharmacological development in the cognitively normal population.

**Result:**

The review highlights high heterogeneity in biomarker‐positive cognitively normal individuals: while isolated amyloid positivity suggests a low progression risk (5–31%)^3^, combined amyloid and neocortical tau pathologies confer a near‐deterministic prognosis^4^. The IWG categorization facilitates biomarker research by aligning profiles with distinct clinical trajectories, enabling the identification of transitional biomarkers. This will further help focus on prognosis biomarkers and identifying mechanisms responsible for resistance or “conductance” of the pathological cascade downstream of β‐amyloidosis. Nosological categorization optimizes pharmacological development by tailoring the risk/benefit ratio of interventions to high‐risk and low‐risk individuals, minimizing risky treatments in low‐risk groups.

**Conclusion:**

The IWG framework bridges biomarker research and clinical outcomes, advancing preclinical AD understanding. By distinguishing “asymptomatic at risk” from “presymptomatic AD,” it fosters biomarker innovation, refines risk stratification, and promotes tailored therapeutic strategies. This framework advances the EuroPAD community's goals, emphasizing the need for individualized interventions and aligning research priorities with clinical and public health imperatives.

**References**:

1 Dubois B, Villain N, Schneider L, et al. Alzheimer Disease as a Clinical‐Biological Construct—An International Working Group Recommendation. JAMA Neurol 2024.

2 Jack CR, Andrews JS, Beach TG, et al. Revised criteria for diagnosis and staging of Alzheimer's disease: Alzheimer's Association Workgroup. Alzheimers Dement 2024; : 1–27.

3 Brookmeyer R, Abdalla N. Estimation of lifetime risks of Alzheimer's disease dementia using biomarkers for preclinical disease. Alzheimer's and Dementia 2018; 14: 981–8.

4 Ossenkoppele R, Pichet Binette A, Groot C, et al. Amyloid and tau PET‐positive cognitively unimpaired individuals are at high risk for future cognitive decline. Nat Med 2022; 28: 2381–7.

